# Differential gene expression revealed with RNA-Seq and parallel genotype selection of the ornithine decarboxylase gene in fish inhabiting polluted areas

**DOI:** 10.1038/s41598-018-23182-z

**Published:** 2018-03-19

**Authors:** C. Vega-Retter, N. Rojas-Hernandez, I. Vila, R. Espejo, D. E. Loyola, S. Copaja, M. Briones, A. W. Nolte, D. Véliz

**Affiliations:** 1 Departamento de Ciencias Ecológicas, Instituto de Ecología y Biodiversidad (IEB), Facultad de Ciencias, Universidad de Chile, Santiago, Chile; 2 Núcleo Milenio de Ecología y Manejo Sustentable de Islas Oceánicas (ESMOI), Departamento de Biología Marina, Universidad Católica del Norte, Coquimbo, Chile; 3 Departamento de Ciencias Ecológicas, Universidad de Chile, Las Palmeras, 3425 Ñuñoa, Santiago Chile; 4 Centro Nacional de Genómica y Bioinformática, Av. B. O’Higgins 340, Santiago, Chile; 5 Departamento de Química, Universidad de Chile, Las Palmeras, 3425 Ñuñoa, Santiago Chile; 6 Carl von Ossietzky University, 26111 Oldenburg, Germany

## Abstract

How organisms adapt to unfavorable environmental conditions by means of plasticity or selection of favorable genetic variants is a central issue in evolutionary biology. In the Maipo River basin, the fish *Basilichthys microlepidotus* inhabits polluted and non-polluted areas. Previous studies have suggested that directional selection drives genomic divergence between these areas in 4% of Amplified Fragment Length Polymorphism (AFLP) loci, but the underlying genes and functions remain unknown. We hypothesized that *B. microlepidotus* in this basin has plastic and/or genetic responses to these conditions. Using RNA-Seq, we identified differentially expressed genes in individuals from two polluted sites compared with fish inhabiting non-polluted sites. In one polluted site, the main upregulated genes were related to cellular proliferation as well as suppression and progression of tumors, while biological processes and molecular functions involved in apoptotic processes were overrepresented in the upregulated genes of the second polluted site. The ornithine decarboxylase gene (related to tumor promotion and progression), which was overexpressed in both polluted sites, was sequenced, and a parallel pattern of a heterozygote deficiency and increase of the same homozygote genotype in both polluted sites compared with fish inhabiting the non-polluted sites was detected. These results suggest the occurrence of both a plastic response in gene expression and an interplay between phenotypic change and genotypic selection in the face of anthropogenic pollution.

## Introduction

Understanding the plastic responses and molecular basis of biological adaptation to environmental changes has become an important goal in environmental ecology. The ability of populations to adapt to these changes is one of the main foci in the field of ecological genomics^1^, which is an important issue considering the increasing habitat degradation that results from human activities. Among the threats produced by human activity, pollution is a major force affecting freshwater systems^2^. This condition can be defined as the result of substances/contaminants entering water bodies and degrading the quality of water^3^. Thus, measurements of water degradation can be used to determine pollution levels. In this context, the detection of changes in gene expression in organisms exposed to diverse pollutants has yielded valuable insights into how organisms react to pollution. For example, the freshwater fish *Fundulus grandis* (Baird and Girard 1853) has a complex genomic response to oil exposure, with 1,070 downregulated and 1,251 upregulated genes^4^. Embryos of *Oryzias melastigma* (McClelland 1839) show differential gene expression related to neurobehavioral defects, mitochondrial dysfunction and the metabolism of proteins and fats after exposure to the organic pollutant Perfluorooctane sulfonate^5^. Gene expression in the liver of the freshwater fish *Lota* (Linnaeus 1758) from Lake Mjøsa (contaminated with organic pollutants) and Lake Losna (a non-polluted lake) was analyzed, and the profile suggested enrichment in the mechanism associated with drug metabolism and increased oxidative stress in organisms living in the polluted Lake Mjøsa^6^. On the other hand, a study of transcriptional responses has revealed part of the mechanistic basis of the pollution tolerance of the freshwater fish *Fundulus heteroclitus*^7,8^.

Despite these steps towards understanding how organisms cope with unfavorable environmental conditions, the relationship between selective forces and phenotypic/genotypic changes remains insufficiently studied. The development of massively parallel Next Generation Sequencing (NGS) facilitates studies on the genomic basis of adaptation even in non-model organisms^9–11^. Genomics and transcriptomics represent complementary methods to identify genes involved in adaptation. Variations in gene expression can be subject to selection^12,13^, and evolutionary novelty can be achieved through changes in transcription levels. Studies of gene expression can document how organisms respond phenotypically, and population genetics studies can reveal alleles under selection that are involved in genetic adaptation.

The Maipo River is one of the most polluted basins in Chile. It is impacted by mining in the Andes Mountains and by factories surrounding the basin^14^. The Maipo River supplies water to 6.7 million inhabitants (40% of the Chilean population) according to the most recent census in 2012 but also receives wastewater from these inhabitants. This basin has experienced water quality degradation and eutrophication^15^, mostly as a product of organic matter from untreated sewage. Studies of fish diversity over the last 30 years have shown a significant reduction in species richness and abundance in this basin^16^, probably associated with habitat degradation and pollution. One species that has shown an important decrease in abundance is the silverside *Basilichthys microlepidotus* (Jenyns 1841), an atherinid endemic to Chile that is considered “vulnerable”^17^. This species inhabits lakes and rivers from 28° to 39°S^18^; it feeds on insect larvae, small invertebrates, filamentous algae and detritus^19,20^. A previous study in the Maipo basin showed the presence of five populations, two inhabiting polluted and three inhabiting non-polluted areas of the river. This pollution categorization was defined using the British Columbia Water Quality Index^21^ based on the attainment of water quality objectives and taking into consideration the natural water quality expected at any given site. Furthermore, Principal Component Analysis indicated that the total dissolved solids, electrical conductivity, chloride, sulfate, molybdenum, ammonium, copper and nitrite were the principal variables accounting for the variance among sites^22^. Water degradation observed in the two polluted sites was the result of different contaminants, such as heavy metals from mining and factories, pesticides from agriculture and dioxins from industrial processes, among others. However, other variables could also be responsible for the differences among sampling sites; for example, river morphology, flow rate, vegetation and bottom morphology. Another study detected that 4% of AFLP loci were most likely under directional selection when comparing individuals inhabiting polluted and non-polluted areas, which suggests that local adaptation takes place^23^. Importantly, different loci were under directional selection in the different polluted sites^23^. This is not surprising because the characteristics of the pollutants along the river are different; a previous work showed that the physicochemical characteristics differ between both polluted sites studied, with clear differences in dissolved oxygen and the concentrations of copper and molybdenum^22^. Thus, it is probable that pollution influences natural populations in different ways, triggering adaptation to the local characteristics of the pollutants. Given the presence of *B. microlepidotus* in polluted and non-polluted areas of the basin and the evidence of signatures of directional selection, the Maipo river basin represents a promising system in which to study genes involved in both plastic and adaptive responses to different environmental conditions.

Thus, the main question of this work was whether environmental pollution is related to phenotypic (gene expression) and/or genotypic selection in natural populations. We hypothesized that the pollution in the Maipo River basin is implicated in a plastic and/or genetic response in *B. microlepidotus*. A transcriptomics approach based on RNA-Seq was used to identify differentially expressed genes by comparing individuals collected from populations inhabiting non-polluted and polluted areas. RNA-Seq analysis was performed using liver samples of *B. microlepidotus* because the liver performs important and complex biological functions that are essential for survival, such as nutrient synthesis, transformation and storage, and endogenous and exogenous substance detoxification^24^. Additionally, the ornithine decarboxylase gene, one of the most upregulated genes in *B. microlepidotus* inhabiting polluted areas, was chosen for an in-depth population genetics analysis to detect evidence of genotype selection.

## Results

### Physicochemical differences among sites

**Figure 1:**
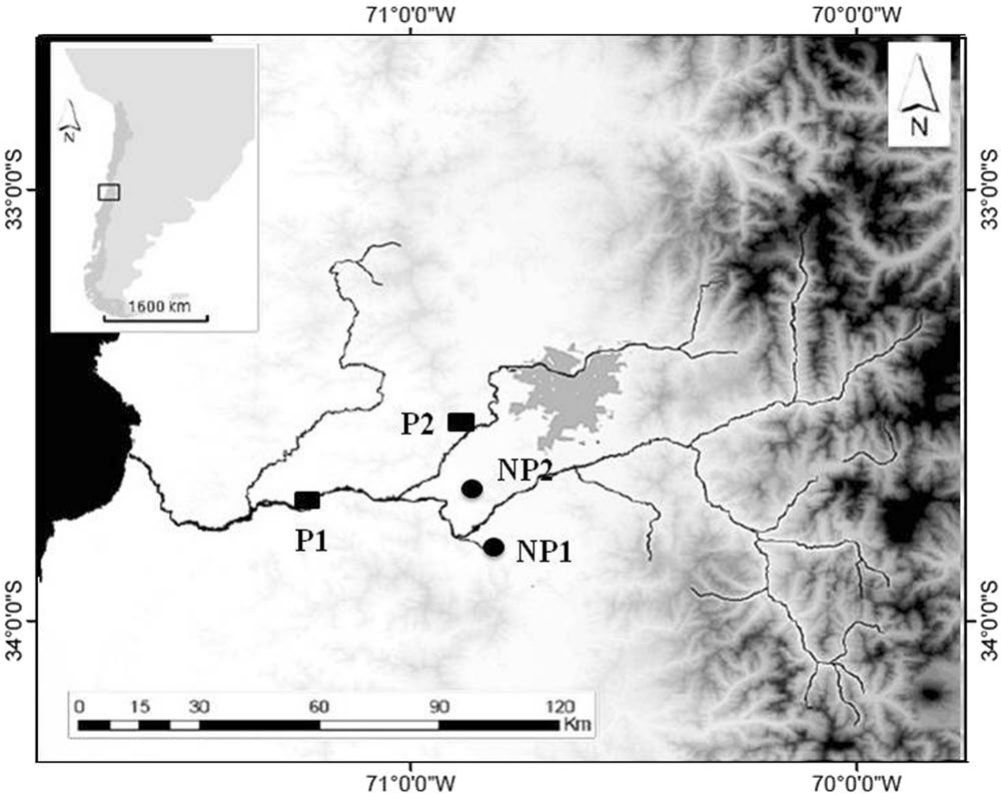
*B. microlepidotus* sampling sites in the Maipo River basin. Circles represent non-polluted sites (NP1 and NP2), and rectangles represent polluted sites (P1 and P2). The map was created with the ArcGIS program version 9.3 (https://www.arcgis.com/features/index.html).

Four sampling sites from a previous study^22^ were chosen for this study: two sites defined as non-polluted: San Francisco de Mostazal (NP1) and Isla de Maipo (NP2); and two others defined as polluted: Melipilla (P1) and Pelvin (P2) (Fig. 1). Based on a population structure study performed previously^22^, we chose four sites that represent independent silverside populations.

**Figure 2:**
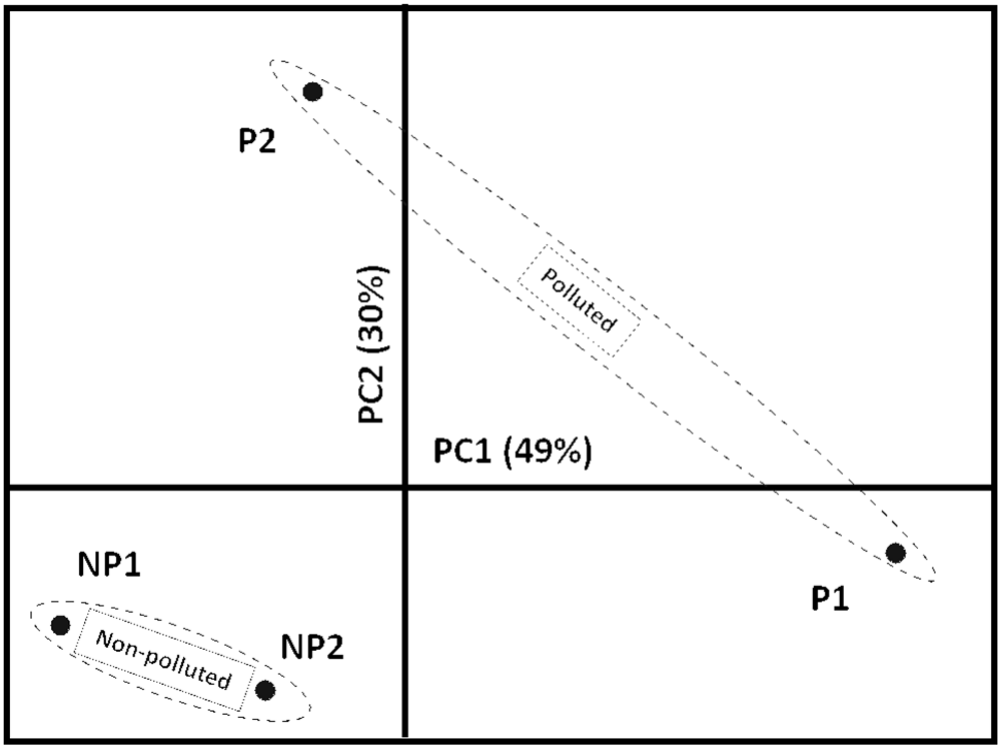
Principal Component Analysis performed with 16 environmental variables measured at the four sampling sites (10 variables measured in water and 6 measured in sediment). The non-polluted group contains: NP1 (San Francisco de Mostazal) and NP2 (Isla de Maipo), and the polluted group contains: P1 (Melipilla) and P2 (Pelvin). PC1 = Principal Component 1; PC2 = Principal Component 2. The ellipse partitions the sites of both conditions, non-polluted and polluted, into two separate groups.

The physicochemical variables measured in water from the four sites included the following: pH, sulfate (SO^2−^_4_), nitrite (NO^−^_2_), ammonium (NH^+^_4_), nitrate (NO^−^_3_), phosphate (PO^3−^_4_), potassium (K^+^), magnesium (Mg^2+^), Dissolved Oxygen (DO), Biological Oxygen Demand (BOD_5_) and sediment characteristics: pH, electrical conductivity (EC), soluble phosphorous (P), percentage of total organic carbon (%TOC), lead (Pb) and zinc (Zn). These variables were analyzed using Principal Component Analysis (PCA). PCA showed similar results to those found previously^22^ among the sites categorized as polluted and non-polluted. Moreover, PCA partitioned the two polluted sites into two separate categories (Fig. 2). The first two components of PCA accounted for 79% of the total variance (Fig. 2), where PC1 (49% of the variance) showed high loadings for two variables measured in the water (NO^−^_2,_ NH^+^_4_) and two in the sediment (P, EC). PC2 (30% of the variance) showed high loadings for_,_ Mg^2+^ and Zn from water samples and pH and Pb from the sediment. Interestingly, two (NH^+^_4_ and NO^-^_2_) out of the eight variables that accounted for most of the variance in this PCA were also observed in a previous work^22^. The raw data (Supplementary Table S2) show that differences between P1 – P2 and NP1 – NP2 are primarily explained by NO^−^_2_, NH^+^_4_, PO^3−^_4_, K^+^ and BOD_5_ in the water and Zn in the sediment, while differences between P1 and P2 could be explained by the concentrations of NO^−^_2_ and NH^+^_4_ measured in the water and P measured in the sediment (Supplementary Table S2).

### Short read and quality filtration, de novo transcriptome assembly and read mapping

A total of 30.15 million reads (4.12 Gb) were obtained from four independent RNA-Seq runs (3 Ion Torrent runs and 1 Ion Proton run). The mean read length in the three Ion Torrent runs was 153.26 bp, and the mean read length in the Ion Proton run was 113.82 bp. After the trimming process, a total of 22.80 million reads (3.16 Gb) was retained for mapping, with a final number of reads per individual ranging from 1.3 million (0.17 Gb) to 3.85 million (0.59 Gb). Considering the genome size of approximately 1 Gb for *B. microlepidotus* and that 1% of the genome corresponding to the transcriptome, the individual coverage ranged from 23 to 68x (mean = 37.44; sd = 11.40). For *de novo* assembly, 14.46 million non-redundant reads (2.13 Gb) were used. A total of 34,786 contigs were longer than 200 bp, with an N50 = 674, and the largest contig contained 6,472 bp. A total of 34,321 non-redundant contigs were finally obtained. The analysis performed with BUSCO, using the Metazoan gene database, revealed that 44.9% of BUSCO genes were “complete”, 35.6% were “fragmented”, and the remaining 19.5% were “missing”, while for the Core Vertebrate Genes dataset, 21.9% of genes were “complete”, 39.5% were “fragmented” and 38.6% were “missing”. In both cases, the quality of the silverside assembled unigenes was comparable to or better than that of most of the transcriptome assemblies reported in a previous work^25^. It is important to note that 11,094 contigs were matched by blast, which corresponds to 83.7% of the transcripts blasted. This Transcriptome Shotgun Assembly project was deposited in DDBJ/ENA/GenBank under the accession number GEVG00000000. The version described in this paper is the first version, GEVG00000000.1.

When the reads for each *B. microlepidotus* were mapped back to the assembled transcriptome, the percentages of mapped reads ranged from 61.27% to 87.25% per individual, with an average of 74.23% (sd = 7.72).

### Differential expression and enrichment analysis

Considering the previous information (different loci under selection in the polluted sites and the physicochemical differences between both polluted sites) and the results obtained from the PCA performed in this study, two different data sets were used for analyses. Dataset A considered contigs that were expressed in the three individuals from the P1 site and expressed in at least 4 of 5 individuals from non-polluted sites. Dataset B was based on contigs that were expressed in all individuals of the P2 site and in at least 4 of 5 individuals from non-polluted sites. There were 10,928 and 12,619 contigs from datasets A and B, respectively. A custom Perl script was used to choose contigs that were expressed in at least 4 out of 5 individuals from non-polluted sites and in at least 5 out of 6 individuals from polluted sites. In total, 13,252 contigs were obtained and used to perform the blast and annotations.

When individuals from P1 (dataset A) and P2 (dataset B) with fish from non-polluted sites were compared, a total of 40 and 36 genes that were differentially expressed were detected, respectively. After removing genes containing outlier data (outliers were defined as individuals in a condition showing expression 1.5% greater than the Interquartile Range (IQR)), 17 and 16 upregulated genes were retained in P1 and P2, respectively. In addition, one downregulated gene was detected at the P1 site (Supplementary Table S3). Most of the annotated genes with the highest FC were associated with the P1 site, corresponding to genes encoding the following: cysteine serine-rich nuclear protein 1 (FC = 24.32), transcription factor jun-b-like (FC = 19.69), phosphoenolpyruvate cytosolic (FC = 16.69), and ornithine decarboxylase (FC = 18.13), while intestinal trypsin, which is involved in the biological processes of digestion and proteolysis, was downregulated (FC = 2.55). The upregulated contigs in datasets A and B were linked to biological processes, such as response to stress, lipid biosynthetic processes, cation transport, negative regulation of cell proliferation or regulation of transcription. As expected, fish sampled in the two polluted sites did not show the same differential gene expression (Supplementary Table S3). This evidence confirms that the physicochemical characteristics and responses of fish in each polluted site are different; therefore, this evidence justifies our analysis that considered the physiological state of the individuals from each site independently.

Enrichment analysis performed for the upregulated contigs in the P2 site showed an overrepresentation of the biological processes and molecular functions involved in apoptotic processes. Biological processes in response to metal ions, autophagic cell death, and the cellular response to glucose starvation were also overrepresented (Table 1). As an example, one of the annotated genes with the highest FC (FC = 19.16) associated with the P2 site, cyclin-dependent kinase inhibitor 1B, is involved in the biological process and molecular function of apoptosis (Supplementary Table S3). In the case of individuals sampled at P1, the upregulated genes showed an overrepresentation of phosphoenolpyruvate carboxykinase (PEPCK) (GTP) activity. Many Gene Ontology (GO) terms of biological processes, molecular functions and cellular components related to microtubules were also overrepresented (Table 1).

**Table 1:** Enrichment analysis performed for genes upregulated in P1 and P2 site. All GO-Terms were over-represented.

GO-ID	Term	Category*	*P*-value
*Enrichment analysis of genes upregulated in P1*			
GO:0005828	kinetochore microtubule	C	1.53e^−3^
GO:0031592	centrosomal corona	C	1.53e^−3^
GO:0043515	kinetochore binding	F	1.53e^−3^
GO:0030981	cortical microtubule cytoskeleton	C	1.53e^−3^
GO:0007020	microtubule nucleation	P	3.05e^−3^
GO:0001578	microtubule bundle formation	P	3.05e^−3^
GO:0051010	microtubule plus-end binding	F	3.05e^−3^
GO:0004613	phosphoenolpyruvate carboxykinase (GTP) activity	F	3.05e^−3^
GO:0004611	phosphoenolpyruvate carboxykinase activity	F	3.05e^−3^
GO:0003854	3-beta-hydroxy-delta5-steroid dehydrogenase activity	F	4.58e^−3^
GO:0046785	microtubule polymerization	P	4.58e^−3^
*Enrichment analysis of genes upregulated in P2*			
GO:0034708	methyltransferase complex	C	1.73e^−4^
GO:0010038	response to metal ion	P	1.22e^−3^
GO:0021589	cerebellum structural organization	P	1.27e^−3^
GO:0021577	hindbrain structural organization	P	1.27e^−3^
GO:0005072	transforming growth factor beta receptor, cytoplasmic mediator activity	F	1.27e^−3^
GO:0048102	autophagic cell death	P	1.27e^−3^
GO:0016505	peptidase activator activity involved in apoptotic process	F	1.27e^−3^
GO:0008656	cysteine-type endopeptidase activator activity involved in apoptotic process	F	1.27e^−3^
GO:0034715	pICln-Sm protein complex	C	1.27e^−3^
GO:0034709	methylosome	C	1.27 e^−3^
GO:0034663	endoplasmic reticulum chaperone complex	C	1.27 e^−3^
GO:0070013	intracellular organelle lumen	C	1.69e^−3^
GO:0043233	organelle lumen	C	1.75e^−3^
GO:0044428	nuclear part	C	1.89e^−3^
GO:0031974	membrane-enclosed lumen	C	1.93e^−3^
GO:0010035	response to inorganic substance	P	2.09e^−3^
GO:0006884	cell volume homeostasis	P	2.54e^−3^
GO:0071850	mitotic cell cycle arrest	P	2.54e^−3^
GO:0043996	histone acetyltransferase activity (H4-K8 specific)	F	2.54e^−3^
GO:0043995	histone acetyltransferase activity (H4-K5 specific)	F	2.54e^−3^
GO:0048188	Set1C/COMPASS complex	C	2.54e^−3^
GO:0046972	histone acetyltransferase activity (H4-K16 specific)	F	2.54e^−3^
GO:0010485	H4 histone acetyltransferase activity	F	2.54e^−3^
GO:0006919	activation of cysteine-type endopeptidase activity involved in apoptotic process	P	3.81e^−3^
GO:0051787	misfolded protein binding	F	3.81e^−3^
GO:0043982	histone H4-K8 acetylation	P	3.81e^−3^
GO:0043981	histone H4-K5 acetylation	P	3.81e^−3^
GO:0004861	cyclin-dependent protein serine/threonine kinase inhibitor activity	F	3.81e^−3^
GO:0042800	histone methyltransferase activity (H3-K4 specific)	F	3.81e^−3^
GO:0016538	cyclin-dependent protein serine/threonine kinase regulator activity	F	3.81e^−3^
GO:0042149	cellular response to glucose starvation	P	3.81e^−3^
GO:0097202	activation of cysteine-type endopeptidase activity	P	3.81e^−3^

^*^P: Biological process; F: Molecular function; C: Cellular component.

### Genetic variability of the ornithine decarboxylase (odc), gene with a high fold change in polluted sites

**Figure 3:**
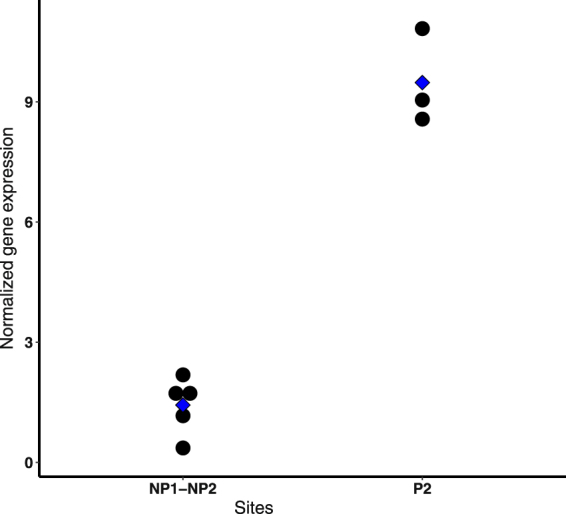
Dotplot showing the normalized gene expression of the *odc* gene in the individuals of the non-polluted sites (NP1-NP2) and the individuals of one polluted site (P2). Each black point represents the normalized gene expression of one individual; the blue point represents the mean of the treatment.

The *odc* gene was significantly upregulated in individuals from P1 in comparison with individuals inhabiting non-polluted areas (FC = 18.13). It is important to note that the *odc* gene also showed increased expression (FC = 6.62) in P2, but this upregulation was not significantly different despite the high difference that both conditions present (Fig. 3) probably because of the high restrictions that we imposed on the analysis.

**Figure 4:**
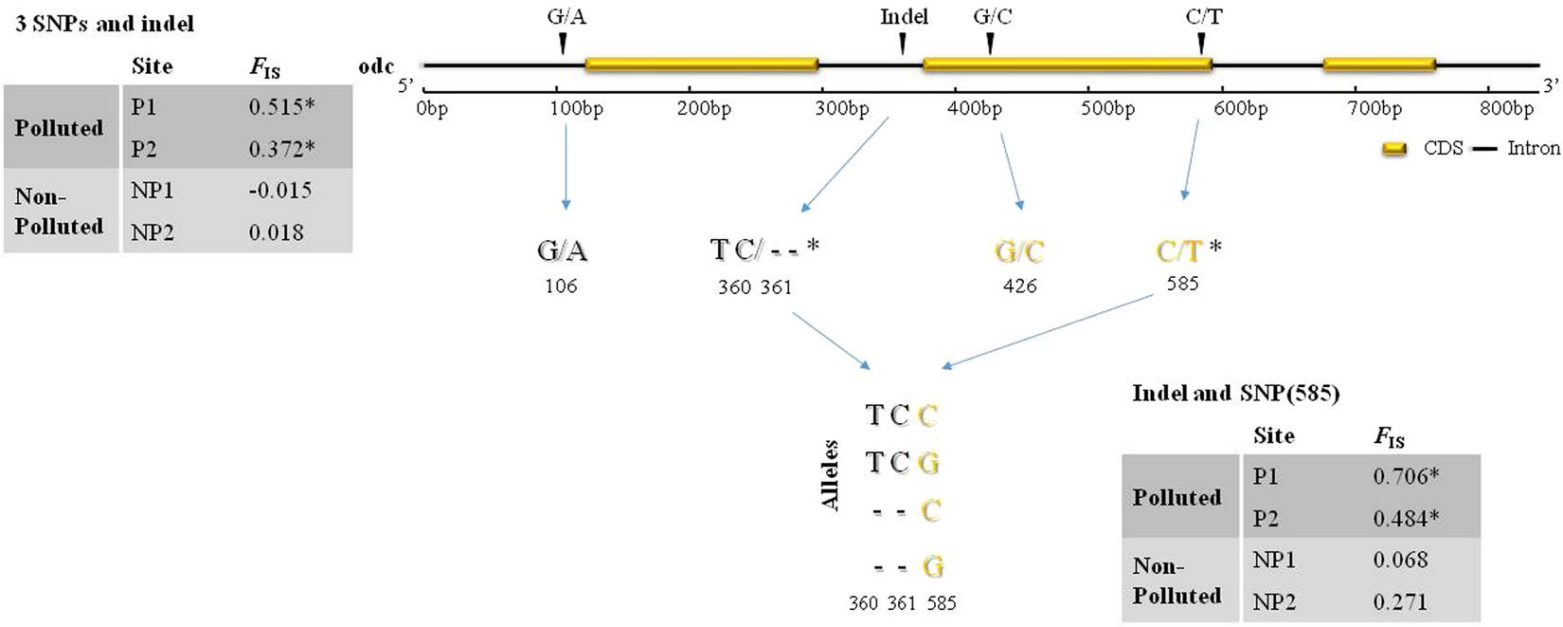
Positions of introns, exons, mutations and indel in the sequenced *odc* fragment. Below the sequence are shown the base change of the mutation and deletion/insertion events. The table located at the left of the figure shows the *F*_IS_ values obtained when the 3 mutations and deletion/insertion events were used. The table located at the bottom shows the *F*_IS_ values obtained for the mutation at position 585 and deletion/insertion events, both showing significant departures from HWE when analyzed separately. Yellow letters indicate SNPs positioned in an exon, while black letters indicate mutations positioned in an intron. *Denotes departures to the HWE; CDS = coding sequence.

The primers designed for the study of the *odc* gene allowed for the sequencing of an 839-bp fragment containing 4 introns (intron A: position 1–122; intron C: 297–375; intron E: 595–675; and intron G: 763–839) and 3 exons (exon B: 123–296; exon D: 376–594; and exon F: 676–762). This fragment was successfully amplified by PCR for all individuals. There were no missing data, suggesting that allele dropout due to PCR failure did not significantly affect our dataset. Six alleles were observed and differentiated by three mutations: one in intron A (A/G in position 106), two positioned in exon D (G/C in position 426; T/C in position 585) and an insertion/deletion event in intron C at position 360–361 (Fig. 4). Both exonic Single Nucleotide Polymorphisms (SNPs) are synonymous substitutions encoding leucine and phenylalanine, respectively. Thus, alleles were constructed considering the complete sequence and were named following the nucleotide present at the positions where the single nucleotide polymorphism was observed. The six alleles detected were: GTCGC, G–GT, GTCGT, ATCGC, GTCCT and G–GC.

All of the sampling sites showed a similar number of alleles; however, the *F*_IS_ index showed strong departures from the Hardy-Weinberg Expectation (HWE) in both polluted sites, but not in the non-polluted sites. These departures were due to the deficit of heterozygotes (Table 2). Estimations of *F*_IS_ performed for each position showed that the insertion/deletion event (located in the intron) and polymorphism T/C at position 585 (located in the exon) explain this departure (Fig. 4).

**Table 2:** Summary of genetic variables estimated from the *odc* gene. n: sampling size, NA: number of alleles, Ho: observed heterozygosity, He: expected heterozygosity, *F*_IS_: inbreeding coefficient and its respective *P* value. *Denotes departures to the HWE.

		n	NA	Ho	He	*F*_IS_	*P*
Polluted	P1	17	4	0.235	0.464	0.515	0.007*
	P2	21	5	0.143	0.220	0.372	0.028*
Non-polluted	NP1	19	4	0.684	0.657	−0.015	0.629
	NP2	19	4	0.632	0.626	0.018	0.543

**Figure 5:**
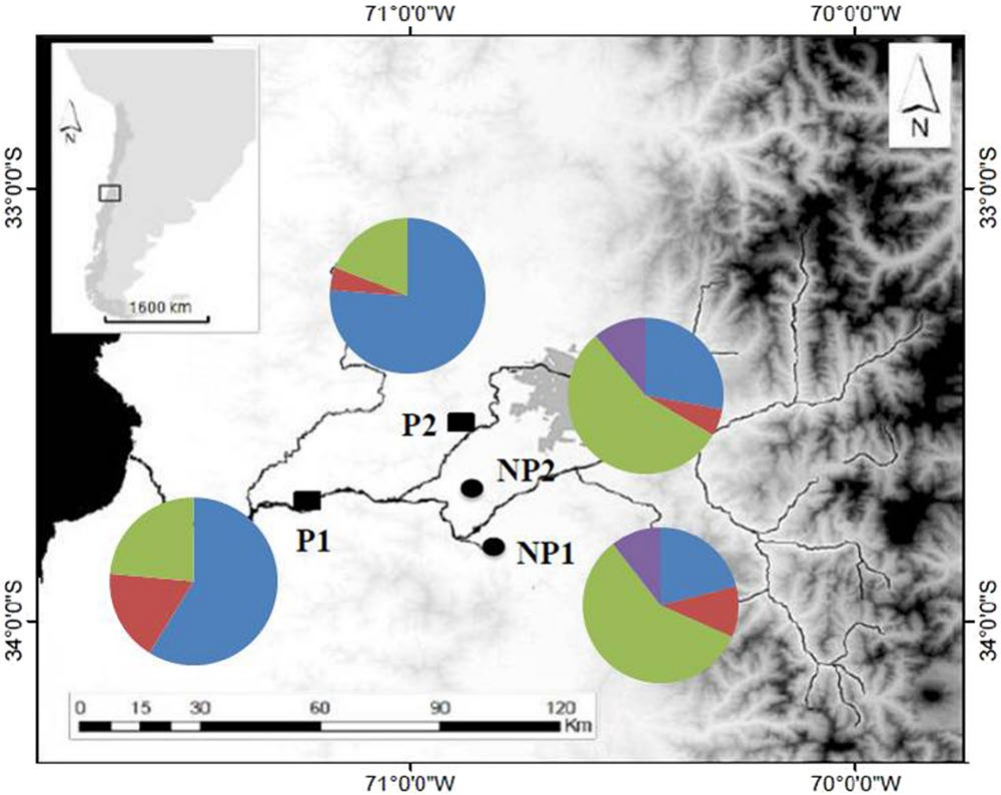
Genotype frequency observed for each sampling site. Non-polluted sites: NP1 (San Francisco de Mostazal) and NP2 (Isla de Maipo); polluted sites: P1 (Melipilla) and P2 (Pelvin). Blue: frequency of the homozygote (GTCGC, GTCGC); red: frequency of the homozygote with the deletion/insertion event (G–GT, G–GT); green: frequency of the heterozygotes with the GTCGC allele (GTCGC, GTCGT), (GTCGC, ATCGC), (GTCGC, GTCCT), (GTCGC, G–GC), and (GTCGC, G–GT); purple: frequency of heterozygotes without the GTCGC allele (G–GC, G–GT), (ATCGC, G–GT), and (GTCCT, G–GT).

To examine the pattern of change, we analyzed the number and frequency of the genotypes considering the complete sequence (including the three mutation sites and insertion/deletion event). A total of 11 genotypes were observed; of these, eight were heterozygotes and 3 were homozygotes. The homozygote (GTCGC, GTCGC) was observed in 26% and 21% of individuals collected from the non-polluted sites (NP1 and NP2, respectively); however, this value increased in individuals inhabiting the polluted sites (59% in P1 and 76% in P2). To quantify this difference, Fisher exact tests were performed to detected differences in the genotype and allele frequencies. For the GTCGC, GTCGC genotype, these analyses showed significant differences between the pooled data of the non-polluted sites with P2 (*P* < 0.001) and P1 (*P* = 0.006). Heterozygotes containing the allele GTCGC showed higher frequencies in non-polluted sites (NP2 = 0.526 and NP1 = 0.579) than polluted sites (P2 = 0.190 and P1 = 0.235), also showing significant differences between the pooled data of the non-polluted sites with P2 (*P* = 0.0124) and P1 (*P* = 0.0411). Interestingly, heterozygotes that do not harbor the GTCGC allele showed a frequency of 0.105 in the non-polluted sites; however, these heterozygotes were absent in the samples obtained from the polluted sites. The change in genotype frequency was also detectable by analyzing the allele frequencies. In this case, the frequency of the GTCGC allele was higher in the polluted sites (P2 = 0.86 and P1 = 0.71) than in the non-polluted sites (NP2 = 0.53 and NP1 = 0.5). The Fisher exact test indicted a significant difference between the non-polluted sites and P2 (*P* < 0.001) and marginal significance for the non-polluted sites and P1 (*P* = 0.0646). Furthermore, the homozygote containing the insertion/deletion event (G–GT, G–GT) had similar frequency values in all of the sites studied (polluted: P2 = 0.048 and P1 = 0.176; non-polluted: NP2 = 0.053 and NP1 = 0.105) (Fig. 5). Finally, the homozygote (ATCGC, ATCGC) was found at a low frequency (0.053) in one site (NP2).

## Discussion

The main goal of this study was to identify differentially expressed genes and detect genotypic selection related to pollution in natural populations of *B. microlepidotus*. Our analysis detected different differentially expressed genes in fish inhabiting both polluted sites. This evidence, together with the differences between both polluted sites observed in PCA, suggests that both sites could be influenced by different types and levels of pollution. Considering that fish showed different physiological responses, we analyzed the polluted sites separately.

Four genes with the highest FC were associated from fish from P1; one of the genes, phosphoenolpyruvate cytosolic, is responsible for the molecular function of PEPCK (GTP) activity. PEPCK is a nucleotide triphosphate (NTP)-dependent enzyme that catalyzes reversible decarboxylation and concomitant phosphorylation of oxaloacetic acid into phosphoenolpyruvate (PEP), once considered to be the rate-controlling enzyme for gluconeogenesis. PEPCK activity and other factors have been suggested to be responsible for fluctuations in gluconeogenesis^26^. Overall, an increase in PEPCK means an increase in the gluconeogenesis process. Gluconeogenesis upregulation has been described in fish exposed to phenol and mercury^27,28^. On the other hand, a decrease in metabolic activity; reduced lipid metabolism, protein biosynthesis, proteolysis, and cellular respiration; and an increase in gluconeogenesis were observed in the livers of individuals of *Danio rerio* exposed to 21-days of starvation^29^. A consideration of the fact that the only gene downregulated in the P1 site is involved in proteolysis raises the possibility that individuals of *B. microlepidotus* inhabiting this site could be suffering from starvation.

The three other genes with the highest FC are possibly related to cancer: cysteine serine-rich nuclear protein and transcription factor jun-b-like are related to tumor suppression and ornithine decarboxylase is related to cancer development. Downregulation of cysteine serine-rich nuclear protein 1 (CSRNP-1) was observed in lung, kidney, liver and colon cancers in its human orthologue gene (AXUD1) compared with the corresponding normal tissues, suggesting that AXUD1 is potentially a tumor suppressor gene^30^. However, mice bred with deficient CSRNP-1 failed to show any increase in cancer^31^. The transcription factor Jun-b-like is a protein of the JUN family that, together with the FOS protein family, are members of the dimeric transcription factor Activator Protein 1 (AP-1). AP-1 has been implicated in different biological processes in cell function^32^, and studies in mice revealed essential functions of AP-1 in controlling liver development, homeostasis, and disease^24^. Specifically, Jun-b has been identified as a negative regulator of cell proliferation, which also upregulates tumor suppressor genes^33^.

Finally, ornithine decarboxylase (*odc*) is the first and rate-limiting enzyme in polyamine biosynthesis, catalyzing the decarboxylation of L-ornithine to putrescine. The polyamine biosynthetic pathway is a critical regulator of cell growth, differentiation, and cell death, and polyamines are involved in nucleic acid packaging, DNA replication, apoptosis, transcription, and translation^34^. Decreasing activity of *odc* in specimens of the frog *Rhinella arenarum* (Hensel) and the plant *Hydrocharis dubia* (Blume) exposed to organophosphorus pesticides and cadmium has been noted^35,36^. Nevertheless, an increase in its activity in the aquatic plants *Potamogeton crispus* (Linnaeus) and *Spirodela polyrhiza* (Linnaeus) exposed to lead has been detected^37,38^. *odc* activation and an increment in polyamine concentration were associated with tumor promotion and progression. The *odc* gene has been suggested to act as an oncogene because its upregulation is essential for cell transformation. Furthermore, its activity has been used as a biological marker for evaluating tumor growth and aggressiveness^39^. Moreover, in the case of our analysis, many GO terms related to microtubules were overrepresented in genes detected as upregulated in the P1 site. Considering that microtubules are essential components for cell division, cell expansion, and cell morphogenesis^40^, determining the real effect of water degradation due to pollution on cell function is of critical importance. On the other hand, enrichment analysis performed for the upregulated genes in P2 showed that GO terms related to autophagic and apoptotic processes and responses to metal ions were overrepresented. We detected one gene (PREDICTED: plasma membrane calcium-transporting ATPase 1-like isoform X5) involved in the molecular function of metal ion binding and another (PREDICTED: zinc transporter 1) involved in the biological processes of zinc ion transport and responses to zinc ions as upregulated in the P2 site. In a previous study, the authors found that the concentrations of Mo and Cu measured in the same sites explained 20% of the total variance of PCA results^22^. Cu becomes toxic with excessive intracellular accumulation and plays a role in initiating the generation of reactive oxygen species and apoptotic processes^41^. Zn can retard the oxidative mechanisms induced by Cu by modulating reactive oxygen species, maintaining an adequate level of metallothionein and reducing Cu through competitive mechanisms; nevertheless, different concentrations of Zn can also block or accelerate apoptotic processes^41^. Given this evidence, it is important to elucidate the type and level of metal pollution in P2 to determine the exact relationship between the metal concentration and apoptotic process. Additionally, a GO term related to the biological processes of the cellular response to glucose starvation was detected as overrepresented in the dataset of upregulated genes. This raises the possibility that individuals of *B. microlepidotus* may suffer from food scarcity at this site, similar to what was inferred for P1. The individuals collected in P2, as with the individuals collected in P1, showed an increased expression of *odc*, presenting a conspicuous pattern of parallel upregulation; although, statistical significance was only reached for P1. As observed in other studies, not all changes in gene expression are adaptive^42^. In some cases, the changes are only a stress response or the result of genetic variation due to genetic drift that does not lead to differential survival. Future research must be performed on this topic to clarify whether this change in gene expression is due to plasticity or adaptive change.

It is important to note that the physicochemical data obtained in a previous work^22^ and those obtained here showed the same differentiation among the sites, indicating a pattern of differences between polluted and non-polluted sites over time. Our study showed high levels of nitrite, phosphates, ammonium and Zn, among others in polluted areas compared with the non-polluted sites. Other studies have described that high levels of these and/or other elements affect the fitness and physiology of fish located in polluted areas^43–45^. Specifically, nitrite is a well-known toxicant in fish and acts as a disrupter of physiological functions, including ion regulatory, respiratory, cardiovascular, endocrine and excretory processes and the oxidation of hemoglobin to methaemoglobin^46^. In our study, P1 had a high nitrite level (0.2 mg/L), which in other studies has been suggested to promote the emergence and development of infectious diseases^46^. On the other hand, high concentrations of phosphates and ammonium drive ecosystem eutrophication^47^, which is the nutrient enrichment of streams and lakes to the point that it promotes explosive growth of aquatic vegetation and phytoplankton, causing several problems, such as a lack of oxygen and a consequent increase in the fish mortality rate. Human activities can increase the rate at which nutrients and organic substances enter aquatic ecosystems from the surrounding areas^47^. Overall, the effects and toxicity of the different stressors on fish depend on multiple variables, such as water quality (e.g., pH, temperature, cations, anions and oxygen concentration), length of exposure, fish species, fish size, age, health, gender, and individual fish susceptibility^46,48–50^. It is important to consider that this is a field study; thus, other elements, such as pesticides from agricultural activity or dioxin products from industrial processes, could also affect the health of the fish. Moreover, due to the nature of this study, it is not possible to relate the observed patterns directly to pollution and to a specific pollutant.

Considering the results of gene expression, the *odc* gene was selected for population genetics analysis. Several results suggest the effects of ongoing selection in the ornithine decarboxylase gene: i) significant departures from the HWE in polluted sites due to a deficit of heterozygotes, ii) the highest frequency of the GTCGC, CTCGC genotype and GTCGC allele in polluted areas, iii) the reduction of heterozygotes containing the GTCGC allele, and iv) the null presence of heterozygotes that do not contain the GTCGC allele in the polluted sites. A similar pattern was observed in the bivalve *Crassostrea angulata*. In this species, the quantity of homozygotes observed for Malic enzymes (EM) and Phosphoglucomutase (PGM) in a population inhabiting a zone with a high concentration of heavy metals was more than that detected in the reference population^51^. In another case, three loci with a metabolic role (mannose-phosphate isomerase, phospho-glucose isomerase and phosphoglucomutase) were studied in a population of the barnacle *Balanus amphitrite* inhabiting a channel with high chemical pollution and in a population inhabiting a site with low pollution (reference population)^52^. The authors found that barnacles inhabiting the polluted area exhibited a significantly higher percentage of the multi-homozygote class compared with the reference population, which signifies lower genetic polymorphisms in the population.

Signals of selection were detected when the analysis was performed with the complete sequence; however, differences were primarily observed in the insertion/deletion in the intron and the mutation located at position 585 in the exon. This result is similar to that observed in a study on *Drosophila melanogaster*, in which haplotypes containing an insertion/deletion in the intron and a synonymous substitution (A/G) in the exon of the *neurofibromin* 1 gene (*Nf*1-insertion-A and *Nf*1-deletion-G) were related to wing size and development time^53^. The authors noted that the haplotype *Nf*1-deletion was associated with a large wing size, while the haplotype *Nf*1-insertion-A was significantly overrepresented in females with shorter development time.

A possible role of gene introns in adaptive evolution has already been noted. Introns can act as enhancers or silencers that modify the expression levels of their host genes. In most studies, these regulatory elements are in the first intron^54^. In the human *odc* gene, several SNPs have been detected. Functional studies indicated that the allele defined by adenine at position +316 is capable of greater *odc* expression than the allele containing guanine at the same position^55^. Overexpression of *odc* has been linked to enhanced cancer susceptibility in animal models^56,57^. To determine the possible effects of *odc* gene selection in *B. microlepidotus*, which could be related to upregulation in polluted sites or to resistance conferred by the GTCGC, CTCGC genotype in these degraded habitats, the complete gene must be analyzed, especially intron 1 and the other exonic regions, because we may not have sequenced important non-synonymous substitutions.

In a field study such as this, it is almost impossible to control all of the variables that affect the population. Despite this, our analysis shows two independent populations of *B. microlepdidotus* with the following patterns: i) overexpressed genes or processes in which they are involved that have been identified as responses to determined pollutants and ii) the same pattern of change in allele frequencies for the *odc* gene. All of these findings suggest that pollutants may have physiological effects on the fish population and represent a possible selective force on *B. microlepidotus* that causes parallel genotypic selection. However, it is necessary to perform further studies at the P1 and P2 sites to further characterize the substances and pollutants present at each site and to clarify the specific effects of pollution on the food availability and health of *B. microlepidotus* as well as to establish the consequences of selective effects on the *odc* gene for silverside fish.

## Materials and Methods

### Sampling sites, sample collection and physicochemical measurements performed at each site

In November 2012, three individuals from each non-polluted site (mean length = 8.95 cm, SD = 3.17) and three from each polluted site (mean length = 6.72 cm, SD = 2.11) were collected; no external lesions or parasites were observed. A description of the basic fish biological characteristics is available in the supplementary information (Supplementary Table S1). Liver tissues were immediately obtained and transported in RNA-later (Life Technologies, Carlsbad, California, United States) to the laboratory and stored at −80 °C until RNA extraction. At the same time, sediment and water samples were collected at each sampling site to determine habitat quality and for comparison with the physicochemical characterizations performed previously^22^ to obtain a temporal view of the habitat quality. In total, three samples of sediment and water were collected at each site. For the details of the water sampling procedure and laboratory measurements, please refer to the previous study^22^. Sediment samples were collected with a plastic shovel from the top 10 cm of the surface sediment zone and stored at 4 °C.

Most of the water quality variables measured previously^22^ were also analyzed in this study, specifically: pH, sulfate (SO^2−^_4_), nitrite (NO^−^_2_), ammonium (NH^+^_4_), nitrate (NO^−^_3_), phosphate (PO^3−^_4_), potassium (K^+^), magnesium (Mg^2+^), Dissolved Oxygen concentration (DO) and Biological Oxygen Demand (BOD_5_).

Sediment samples were dried in polyethylene trays at room temperature and sieved. Two fractions were obtained from this procedure: the coarse fraction and fine fraction, wish particle sizes of less than 2 mm and 0.063 mm, respectively. The variables measured were: pH, electrical conductivity (EC), soluble phosphorous (*P*), percentage of total organic carbon (%TOC), lead (Pb) and zinc (Zn). EC and pH were determined by potentiometric methods, %TOC was determined by the gravimetric method, and water-soluble phosphorus was measured by the Olsen method^58,59^. For Pb and Zn, a pseudo-total fraction was obtained by digesting 1 g of sediment with 10 mL of nitric acid (65% Suprapur Merck) in a high-resolution microwave oven (MarsX press) based on EPA method 3051.

To determine differences among sites, Principal Component Analysis (PCA) was performed using all of the variables measured in both water and sediment. In this analysis, the factor loadings were used to determine the contribution of the variables to the first two principal components. PCA analysis was performed using the ade4 library implemented in R software^60^.

All of the protocols of this study were approved by the ethics committee of the Universidad de Chile and complied with existing laws in Chile (Resolución Exenta No. 3329 Subsecretaria de Pesca).

### RNA extraction and sequencing

Total RNA from liver samples was extracted using the PureLink^TM^ RNA Mini Kit (Ambion) following the manufacturer’s instructions; the quality and quantity of the total RNA were measured using an Agilent Model 2100 Bioanalyzer, and samples showing a RIN >7 were selected for the following process. Samples were stored in RNase-free water supplemented with Superase-In^TM^ RNase Inhibitor (Ambion, Austin, TX, USA) at −80 °C. Total RNA was subsequently enriched for polyA mRNA using the MicroPoly (A) Purist^TM^ kit (Ambion, Austin, TX, USA). Due to the low RNA extraction, an individual from NP1 was excluded from the analysis. Thus, mRNA from 11 samples was used to prepare 11 separate barcoded libraries with the Ion Total RNA-Seq Kit v2 (Life Technologies). These samples were sequenced in a total of four runs, three in the Ion Torrent using an Ion 318 chip and the fourth in the Ion Proton with the PI chip. Library construction and sequencing were performed by OMICS Solutions (Santiago, Chile). The same RNA-Seq data were used to perform the *de novo assembly* and to assess gene expression.

### Short read, quality control and de novo assembly

This part of the analysis was similar to that described in a previous work^61^. Briefly, short read and quality filtration were performed with PRINSEQ^62^ and TRIMMOMATIC^63^ software. TRIMMOMATIC was used with a sliding window trimming of size 10 and a threshold of average quality of 15. Reads shorter than 60 bp were removed. PRINSEQ was used to remove reads with low mean phred scores (<Q15), PolyA and PolyT tails at the 5′ and 3′ ends, reads shorter than 60 bp and to trim reads longer than 250 bp to this length as well as also to remove repeated reads from the set used for the *de novo* assembly. The *de novo* assembly was performed using the MIRA assembler^64^, which is based on an overlap layout consensus (OLC) algorithm. The overlap between each pair of reads was computed and compiled into a graph, where each node represents one short read and the edge between two nodes indicates that the two short reads have overlapping sequences. After several steps of simplifying the overlap graph by removing the transitive nodes and edges, a chain of nodes elicits the sequence of a contig. For our data analysis, the minimum number of reads per contig was set to 10. To create a dataset of non-redundant contigs, CD-HIT software (http://weizhong-lab.ucsd.edu/cd-hit/) was used with an identity cut-off threshold of 90%. “Bench-marking universal single-copy orthologs” (BUSCO) software (v2/v3) was run in gVolante^65^ to identify core genes, which is a set of single copy genes that are highly conserved among eukaryotes and thus expected to be present in a complete assembly^25^. BUSCO analysis was performed using the Metazoan and Core Vertebrate Genes datasets, which consist of 978 and 233 single-copy orthologs, respectively.

### Mapping of reads and contigs annotations

To test for differentially expressed genes, individual reads for each *B. microlepidotus* were mapped back to the assembled transcriptome using the alignment program TMAP (http://github.com/iontorrent/TMAP/tarball/tmap.0.3.7). The number of aligned reads with each contig for each sample was estimated by IdxStats command of SAMtools^66^.

Considering the results of the environmental analysis that showed more differences between both polluted sites than between both non-polluted sites (Fig. 2) and the results of previous works, a custom Perl script was used to create two datasets of contigs to perform different comparisons. The first dataset considered the contigs that were expressed in all of the individuals of the P1 site and expressed in at least 4 of the 5 individuals from the non-polluted sites (NP2 and NP1 together) (dataset A), while dataset B was based on contigs that were expressed in all of the individuals of the P2 site and expressed in at least 4 of the 5 individuals from the non-polluted sites.

All of the transcripts that were expressed in at least 4 out of 5 individuals from the non-polluted sites and in at least 5 out of 6 individuals from the polluted sites were blasted using the blastx function of the Blast2GO software^67^. The blastx^68^ function was performed with an E-value score of 1.0E^−06^. To allocate gene ontology (GO) terms to each annotated sequence, successful blast hits were mapped and annotated using Blast2GO with the annotation cut-off threshold set to 55 and GO level weighting set to 5 as recommended by the authors of the software. The basic annotation obtained by retrieving the GO terms from blastx matches was enriched and refined using InterProScan, implemented in Blast2GO software.

### Differential expression and enrichment analysis

Count data from the two contigs datasets were used as the input for the EdgeR analysis^69^ implemented in MultiExperiment Viewer software (http://www.tm4.org/mev.html). Differential expression was tested using a 10% false discovery rate (FDR) between: i) individuals from P1 and individuals from the non-polluted sites (dataset A) and ii) individuals from P2 and individuals from the non-polluted sites (dataset B). We discarded contigs that contained outliers that were 1.5% greater than the Interquartile Range (IQR) based on the boxplot generated in R software^60^. To test if any of the GO terms was significantly overrepresented, one-tailed Fisher’s exact test with a *P*-value of 0.005 implemented in Blast2GO was used. This test was performed for both upregulated and downregulated contigs detected by EdgeR: i) from P1 when the individuals of this site were compared with the fish from the non-polluted sites and ii) from P2 when the individuals of this site were compared with the fish from the non-polluted sites; in both cases the comparison was performed against all the contigs contained in the dataset.

### Sequencing of ornithine decarboxylase (odc)

After RNA-Seq analysis, we identified the most upregulated genes obtained after statistical analysis. Of these genes, ornithine decarboxylase (*odc*) showed a high rate of upregulation in the individuals from P1 (polluted site) compared to the individuals collected in the non-polluted sites, and it also showed a high rate of expression (but not significant) in individuals from P2 compared to the individuals of the non-polluted sites. To detect differences in genotype frequencies, the *odc* gene was sequenced from the same individuals used in a previous work of population genetic structure^22^; 19 individuals of NP2, 19 of NP1, 21 of P2 and 17 of P1 were included. The primers for the *odc* gene were designed using the mRNA sequence of the contig obtained from the RNA-Seq analysis. The “primer-BLAST” tool from Genbank (http://www.ncbi.nlm.nih.gov/tools/primer-blast) was used to design the forward and reverse primer with an annealing temperature of 60 °C. The primers were: Forward primer, 5′ CTT TGG CAT CCC TGG GAA CT and reverse primer, 5′ GAG GGC TGG GTT GAT TAC TG. The PCR reaction mixture had a final volume of 25 µL that included 1 × PCR buffer (Invitrogen), 3.2 nM of MgCl_2_ (Invitrogen), 5 pmol of forward and reverse primers (50 ng/L) (Applied Biosystems), 0.2 U/µL of dNTPs (2.5 mM) (Invitrogen), and 0.1 U/µL of Platinum Taq Polymerase (Invitrogen). To this mixture, 1.5 µL of template DNA (65 ng/ µL) was added. The PCR reaction involved a denaturing step at 94 °C for 3 min, followed by 30 cycles at 94 °C for 30 s, 60 °C for 90 s, and 72 °C for 90 s with a final elongation step at 72 °C for 10 min. PCR products were examined in 1.5% agarose gels and were sequenced by Macrogen, Inc. (www.macrogen.com), in both senses. Sequences were aligned in Proseq software^70^. *odc* is a nuclear gene, so analyses of both forward and reverse sequences were required to clearly detect the heterozygotes. In this sense, consistent half size double picks were considered to be a valid mutation. Insertion/deletion events (indel) were confirmed manually. Due to the presence of three mutations in the sequences, the Phase method in DnaSP v.4.9 software^71^ was used to detect the correct sequence of each allele. The number of alleles per locus and expected (He) and observed (Ho) heterozygosities were estimated using Genetix software^72^. A total of 5,000 allele permutations were used to test for departures from Hardy Weinberg Equilibrium (HWE). The consensus sequence was analyzed and annotated for putative coding vs. non-coding regions based on comparisons between cDNA obtained from RNA-Seq and cDNA and genomic DNA published sequences from other species. The sequences were published in GenBank with the accession numbers: KX608522 to KX608527.

### Data accessibility

Transcriptome shotgun assembly: GEVG00000000 (VERSION GEVG00000000.1) Bioproyect: PRJNA326687. BioSample: SAMN05290829 - SAMN05290839. Sequence Read Archive: SRR3730243 - SRR3730264. *odc* sequences: GenBank accession numbers KX608522 - KX608527.
